# Integrative annotation scores of variants for impact on RNA binding protein activities

**DOI:** 10.1093/bioinformatics/btae181

**Published:** 2024-04-18

**Authors:** Jingqi Duan, Audrey P Gasch, Sündüz Keleş

**Affiliations:** Department of Statistics, University of Wisconsin, Madison, WI, 53706, United States; Laboratory of Genetics, University of Wiconsin, Madison, WI, 53706, United States; Center for Genomic Science Innovation, University of Wisconsin, Madison, WI, 53706, United States; Department of Statistics, University of Wisconsin, Madison, WI, 53706, United States; Center for Genomic Science Innovation, University of Wisconsin, Madison, WI, 53706, United States; Department of Biostatistics and Medical Informatics, University of Wisconsin, Madison, WI, 53706, United States

## Abstract

**Motivation:**

The ENCODE project generated a large collection of eCLIP-seq RNA binding protein (RBP) profiling data with accompanying RNA-seq transcriptomes of shRNA knockdown of RBPs. These data could have utility in understanding the functional impact of genetic variants, however their potential has not been fully exploited. We implement INCA (Integrative annotation scores of variants for impact on RBP activities) as a multi-step genetic variant scoring approach that leverages the ENCODE RBP data together with ClinVar and integrates multiple computational approaches to aggregate evidence.

**Results:**

INCA evaluates variant impacts on RBP activities by leveraging genotypic differences in cell lines used for eCLIP-seq. We show that INCA provides critical specificity, beyond generic scoring for RBP binding disruption, for candidate variants and their linkage-disequilibrium partners. As a result, it can, on average, augment scoring of 46.2% of the candidate variants beyond generic scoring for RBP binding disruption and aid in variant prioritization for follow-up analysis.

**Availability and implementation:**

INCA is implemented in R and is available at https://github.com/keleslab/INCA.

## 1 Introduction

Genome-wide association and exome sequencing studies for rare diseases yield large numbers of potentially causal single nucleotide variants (SNVs). Many tools are available for annotating variants based on genomic features, as reviewed by Hebbar and Sowmya (2022). Furthermore, additional methods with distinct focal points have emerged, such as prioritizing the regulatory elements and predicting the impact of variants, as discussed by Rojano *et al.* (2019). Despite this progress, a limited number of studies exploit epigenomic data from RNA-binding proteins (RBPs). SeqWeaver (Park *et al.* 2021) is trained on RBP CLIP profiles to predict the impact of variants on RBP binding. However, relying solely on SeqWeaver scoring yields hundreds to thousands of variants with indistinguishable scores. To introduce specificity to the scoring by SeqWeaver, we devised INCA. INCA calibrates the SeqWeaver scores by utilizing the variants reported as pathogenic in ClinVar (https://www.ncbi.nlm.nih.gov/clinvar) and aggregates these with cell line level eCLIP-seq and transcriptome data from the ENCODE project (Luo *et al.* 2020). INCA enhances variant scoring for RBP impact with auxiliary data, thereby accentuating the impact of variants on RBP interactions with the DNA.

## 2 Materials and methods

**Figure 1.**
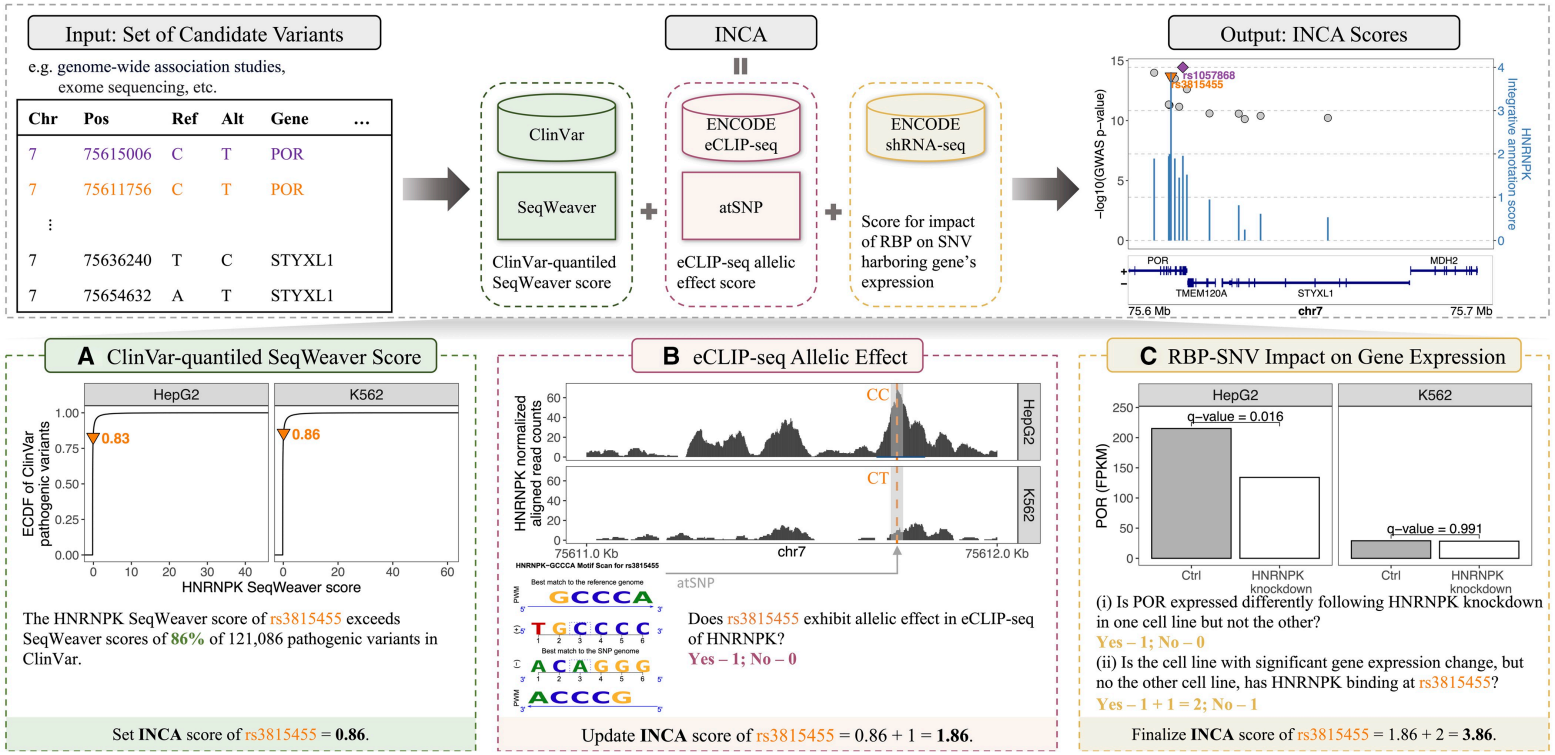
INCA pipeline illustration for lead SNV (in purple) and its LD partners. INCA scores of variants are integrated over three modules. (A) ClinVar-quantiled SeqWeaver scores based on SeqWeaver and ClinVar data. (B) eCLIP-seq allelic effect scores based on ENCODE eCLIP-seq data. Vertical dashed line marks the variant location. (C) Scores for the impact of RBPs on SNV harboring gene’s expression based on ENCODE shRNA-seq data. INCA promotes LD partner rs3815455 with a score of 3.86 over the lead SNV with a score of 1.96.

Figure 1 presents an overview of INCA with an application to 388 lead SNVs for the blood lipid trait triglycerides (TGs) (Graham *et al.* 2021). The selection of TG to showcase INCA is based on two key considerations: (i) K562 (a human erythroleukemia cell line) and HepG2 (a human liver cancer cell line) are the cell lines with the most complete RBP profiling, and (ii) a heritability enrichment analysis using the stratified linkage disequilibrium (LD) score regression with genome-wide association study (GWAS) summary statistics identifies TG-specific enrichment of heritability in regions surrounding genes with the highest specific expression in liver (Finucane *et al.* 2018). This INCA application utilizes: (i) genotypes of K562 and HepG2 cell lines along with 37 RBP eCLIP-seq datasets from each; (ii) 37 accompanying RNA-seq experiments of wild type (WT) and RBP shRNA knockdown conditions; (iii) 121,086 pathogenic SNVs from ClinVar, scored with SeqWeaver. Applications of INCA with other epigenome datasets are discussed in Supplementary Section S5.

### 2.1 Input

INCA takes as input a variant file with the following fields: Chr, Pos, Start, End, Ref, Alt, and Gene. It then derives three scores for the effect of each variant on each RBP activity using available cell lines (K562 and HepG2 for this implementation): (i) ClinVar-quantiled SeqWeaver scores; (ii) a score of allelic effect on RBP binding derived from a pre-computed library of RBP eCLIP-seq experiments (Van Nostrand *et al.* 2020) (Supplementary Section S5); and (iii) a gene impact score for the gene that the SNV is proximal to based on a pre-computed library of accompanying RNA-seq experiments (wild type and RBP knockdown by shRNA).

We first scored all the 121 086 pathogenic SNVs from ClinVar with SeqWeaver. Then, we transformed each input variant’s SeqWeaver score to depict the proportion of ClinVar variants with a score lower than that of the variant’s, generating ClinVar-quantiled SeqWeaver scores. As an illustration, Fig. 1A displays that the SeqWeaver scores of SNV rs3815455 for RBP HNRNPK surpass those of the 86% of pathogenic variants in the K562 cells and 83% in the HepG2 cells. Further elaboration on these scores is available in Supplementary Section S1.

The quantification of allelic effects involves a comparison of eCLIP-seq data for an RBP across two distinct cell lines/types, where one cell line carries the variant allele at the locus of interest, and the other cell line does not. For example, at the locus of SNV rs3815455, we observe a homozygous CC genotype in HepG2 and a heterozygous CT genotype in K562. The GWAS study (Graham *et al.* 2021) suggests that the risk allele for SNV rs3815455 is T which matches the K562 allele and is different from the HepG2 allele. Thus, we quantify the allelic effect of SNV rs3815455 on the RBP HNRNPK by comparing its eCLIP-seq profiles in HepG2 and K562 cells. Specifically, eCLIP-seq allelic effect score quantifies if the SNV resides in a eCLIP-seq enriched region in one cell line but not the other and, hence, exhibiting allelic variation between the lines. Supplementary Section S2 provides further implementation details of the allelic effect score.

Figure 1B displays the eCLIP-seq profiles of RBP HNRNPK in both cell lines and yields that SNV rs3815455 resides in a peak in HepG2 cells with the reference allele C, whereas this genomic location does not exhibit significant binding by HNRNPK in K562 cells with the variant allele T as determined by an IDR analysis (Luo *et al.* 2020). *In silico* motif analysis with atSNP (Zuo *et al.* 2015) provides further support for genetic disruption of the motif (Supplementary Section S3).

## 3 Results

We applied INCA to 388 lead SNVs from a TG GWAS (Graham *et al.* 2021). Of these, 343 have at least one LD partner (LD $R^{2}\;\geq \;0.7$), resulting in a total of 14 945 SNVs of interest (58.4% with SeqWeaver scores). Over a quarter of 183 lead SNVs with a score were bested by >50% of their associated LD variants, highlighting the need for refining SeqWeaver score specificity. Among the 160 lead SNVs without a SeqWeaver score, 17 SNVs (10.6%) have over 10% of their LD partners scoring better than the median score of the ClinVar pathogenic SNVs (Supplementary Fig. S5). Among the 14 945 variants, 33.7% match the genetic variation between HepG2 and K562. INCA scoring of these reveals that LD partners of the 12 lead SNVs (3.5%) have a score greater than 2 (out of 4 total, Supplementary Table S5). On average, 46.2% of a lead SNV’s LD partners receive an INCA score greater than 1 (Supplementary Fig. S6A). Out of the 343 lead SNVs, 32.9% have over 50% of their LD partners scoring higher than their own INCA score (Supplementary Fig. S6B). Collectively, INCA provides 53.8% reduction in the set of variants to follow up. Interestingly, rs3815455 (an LD partner of the lead SNV rs1057868), has a markedly higher INCA score (Fig. 1) and is independently implicated as significantly associated with urinary biomarkers which harbor high genetic correlations with lipid traits (Zanetti *et al.* 2019).

In summary, we introduced INCA as a multi-step process to evaluate the potential of variants for disrupting RBP function by leveraging under-utilized data sources. We expect INCA to be useful for drilling down on annotation of genetic variants with existing epigenomic data, not limited to RBPs. An application using TF ChIP-seq is detailed in Supplementary Section S7, and additional available data resources for evaluating variants using INCA are listed in Supplementary Section S8.

## Supplementary Material

btae181_Supplementary_Data
