# Supplementary material for: Integrative annotation scores of variants for impact on RNA binding protein activities
Source: Bioinformatics. 2024 Apr 18;40(4):btae181. doi: 10.1093/bioinformatics/btae181 (PMC11042904; doi:10.1093/bioinformatics/btae181)
Supplement: btae181_Supplementary_Data [file btae181_supplementary_data.pdf]

Supplementary Materials for "Integrative annotation scores of variants for impact on RNA binding protein activities"

Jingqi Duan[1], Audrey P. Gasch[2,3], and Sündüz Keleş[1,3,4] *

[1]Department of Statistics, University of Wisconsin-Madison, Madison, WI, 53706, USA,
[2]Laboratory of Genetics, University of Wisconsin-Madison, Madison, WI, 53706, USA,
[3]Center for Genomic Science Innovation, University of Wisconsin-Madison, Madison, WI, 53706, USA,
[4]Department of Biostatistics and Medical Informatics, University of Wisconsin-Madison, Madison, 53706, WI, USA.
Corresponding author: *keles@wisc.edu

1 ClinVar-quantiled SeqWeaver scores

SeqWeaver (Park *et al.*, 2021) scores each given variant for its impact on binding of an RBP, compared to the reference allele at the SNV position. The scoring is based on a model pre-trained on the eCLIP-seq data of the RBP. While the score reported for each variant-RBP combination is useful for ranking variants for the impact of specific RBP, thresholds for interpreting these scores are not established. To investigate the general characteristics of SeqWeaver scores, we applied SeqWeaver to 125,774 pathogenic single nucleotide variants from ClinVar (`https://www.ncbi.nlm.nih.gov/clinvar`) and obtained SeqWeaver scores of 121,086 variants for 65 distinct RBPs with a trained SeqWeaver model (42 in HepG2 and 51 in K562 cells). Fig. S1 displays the empirical cumulative distributions of SeqWeaver scores of 121,086 pathogenic ClinVar variants for HNRNPK (in HepG2 and K562) and SF3B4 (in K562) and highlights that the interpretation of a score of 1 would differ depending on the RBP. For instance, a SeqWeaver score of 1 for HNRNPK in HepG2 or K562 surpasses 96% of pathogenic variants, indicating a relatively high score. However, a score of 1 for SF3B4 in K562 only exceeds 68% of pathogenic variants, which would be considered moderate. To address this limitation and make these scores comparable, INCA transforms SeqWeaver score of a variant to represent proportion of pathogenic ClinVar variants that have scores smaller than that of the given variant. This is under the assumption that most ClinVar variants will not affect RBP activities. We refer to this transformed score as the *ClinVar-quantiled SeqWeaver score*. In the case of multiple SeqWeaver scores for the same RBP-variant based on multiple trained SeqWeaver models of the RBP, the maximum score is reported to capture the predicted upper bound for the impact.

2 Allelic effect scores from eCLIP-seq profiles

For each variant with an allelic difference between the cell lines (i.e., one cell line carries the variant allele at the variant locus, and the other cell line does not), INCA computes a score of allelic effect on RBP binding derived from a pre-computed library of RBP eCLIP-seq experiments (Van Nostrand *et al.*, 2020). This score is set to 1 if (1) the variant of interest resides within a binding region of the RBP as defined by an IDR peak in one cell line, but not in the other cell line, or (2) the variant of interest resides in a region of significant enrichment with respect to the normalized read counts in one cell line, but not the other cell line. Setting (1) directly utilizes the IDR peaks reported by the ENCODE project (Luo *et al.*, 2020). Setting (2) aims to capture the enrichment in the eCLIP-seq experiments compared to control experiments that might be missed by the stringent IDR thresholding process (Hitz *et al.*, 2023). A region is labelled as significantly enriched in normalized read counts (i.e., region's aligned read counts normalized by taking into account the control counterparts of the eCLIP-seq experiments and the differences in the sequencing depths) if its signal value, i.e., measurement of overall (usually, average) enrichment for the region, exceeds the median of the signal values of the reported IDR peaks. Our implementation allows the user to

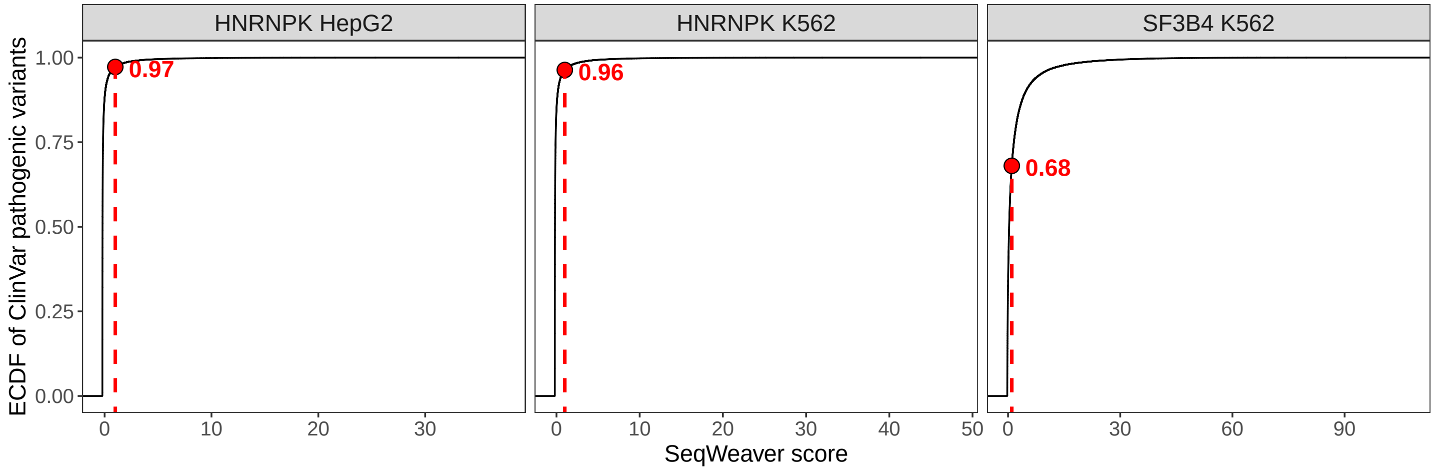

Figure S1: Empirical cumulative distribution of SeqWeaver scores for RBPs HNRNPK and SF3B4 across 121,086 ClinVar pathogenic variants. The quantile that the score of 1 (marked by the vertical dashed line) corresponds to is marked for each RBP. A SeqWeaver score of 1 for HNRNPK in HepG2 or K562 cells surpasses the scores of 96% of the pathogenic variants, indicating a relatively high score. However, a score of 1 for SF3B4 in K562 cells only exceeds 68% of those of pathogenic variants', which would be considered moderate.

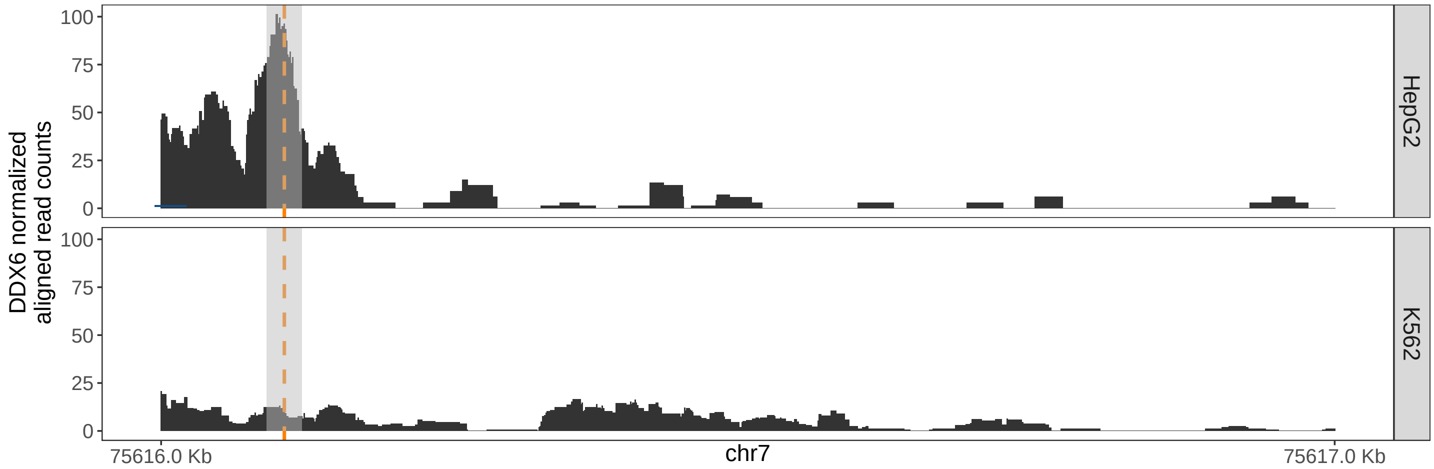

Figure S2: Example of allelic effect score of 1 from setting (2). While SNV rs17685 does not reside within an IDR peak in the HepG2 cell line, its genomic location exhibits marked enrichment of normalized read counts in HepG2 compared to K562 cells.

adaptively choose other thresholds, i.e., 60%, than the median. Fig. 1(B) of the main text displays that the SNV rs3815455 resides within an IDR peak in HepG2 cell line with the reference allele C and exhibits lower normalized read counts in K562 cell line with the variant allele T. This observation suggests that RBP might be interacting with the RNA containing the C allele but disrupted in interaction with the RNA containing the variant with T allele. Consequently, the SNV rs3815455 C>T may cause the loss of RBP binding, specifically, leading to the disruption of HNRNPK binding to the SNV harboring gene POR. Hence, the allelic effect is considered as the loss of RBP binding, and INCA assigns a score of 1 to this SNV. As an example of setting (2), Fig. S2 highlights that while the SNV rs17685 does not reside within an IDR peak in HepG2 cells, this region exhibits marked enrichment of normalized read counts in HepG2 compared to K562. Consequently, INCA also assigns an allelic effect score of 1 to this variant.

Table S1: Composition of the RBP motif library compiled for `atSNP`.

Database	# of motifs	# of RBPs
ATtRACT	1,198	159
CISBP-RNA	140	89
MEME Suite Motif Databases	98	98 (CISBP-RNA)
	102	80 (RNA, Ray2013)
RBPDB	53	29
RBPmap	276	176

3 *In silico* motif analysis of RBP binding

INCA augments the scores of variants with allelic effect with an *in silico* motif analysis by leveraging the R package `atSNP` (Zuo *et al.*, 2015). `atSNP` assesses whether the variant is likely to disrupt or enhance binding of the RBP based on its impact on the actual binding sequence. While `atSNP` was originally developed for DNA binding proteins, i.e., transcription factors, we updated its motif libraries to encompass binding sites of RNA binding proteins. Specifically, we compiled position weight matrices (PWMs) for 1,217 motifs across 196 RBPs from the following databases: ATtRACT (Giudice *et al.*, 2016), CISBP-RNA (Ray *et al.*, 2013), the MEME Suite Motif Databases (Bailey *et al.*, 2015), RBPDB (Cook *et al.*, 2011), and RBPmap (Paz *et al.*, 2014). Table S1 presents detailed information regarding the number of motifs and RBPs from each database. Fig. 1(B) of the main text displays the results for application of `atSNP` with this motif library to SNV rs3815455, which changes reference C allele to a T allele. We observe that with the reference allele C, the sequence that resides in the SNV location closely matches the binding sequence GCCCA of the RBP HNRNPK. This match appears to be disrupted by the SNV T allele. This correlates with the observed loss of binding in the K562 cell line with the variant allele compared to the HepG2 cell line with the reference allele (eCLIP-seq tracks of Fig. 1(B) of main text).

4 RBP-SNV impact on gene expression

Differential gene expression analysis with shRNA knockdown of RBPs allows the identification of genes directly or indirectly regulated by the RBP. Genes that harbor RBP binding sites (i.e., peaks from the corresponding eCLIP-seq experiments of the RBP) and exhibit significant changes in expression upon RBP knockdown are likely to be direct targets of the RBP. INCA scores the variant harboring genes to assess whether the gene might be a target of the RBP, binding of which the SNV might be impacting. Specifically, leveraging the differential expression analysis results of RNA-seq experiments of RBP knockdowns by shRNA and wild type conditions, the RBP-SNV gene impact score evaluates if (i) the gene displays significant changes in expression upon RBP knockdown in one of the cell lines, and (ii) the cell line with the expression change has an RBP peak covering the location of the variant. When both conditions hold only in one of the cell lines, it broadly implies that the encoded transcript is a binding-target of the RBP with expression consequences in that cell line, leading to a INCA score of 2, and resulting in a score of 1 if only the former condition holds. Table S2 illustrates all possible scenarios for (i) and (ii) and their corresponding scores for RBP-SNV gene impact. Fig. 1(C) of the main text illustrates that POR is expressed robustly in both the HepG2 and the K562 cells. In the HepG2 cell line with the reference allele C and a HNRNPK eCLIP-seq peak overlapping the variant location, knocking down HNRNPK leads to significant changes in POR expression (q-value of 0.016), suggesting that HNRNPK has a regulatory effect on POR. However, in the K562 cell line with the variant allele T and without significant HNRNPK eCLIP-seq enrichment at the variant location, HNRNPK knockdown does not have any discernible effect on gene expression (q-value of 0.991), indicating that the regulatory relationship between HNRNPK and POR may already have been disrupted due to the variant.

5 eCLIP-seq library for RBPs

To enhance the computational efficiency, we developed a pre-computed library containing aligned reads and peak information from the ENCODE eCLIP-seq collection of 223 RBPs in HepG2 and K562 cell lines. This library provides convenient access

Score for RBP-SNV impact on gene expression	(ii) RBP peak at variant location			
	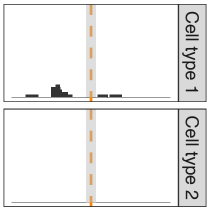	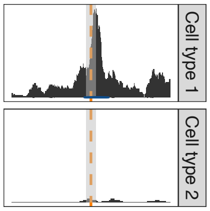	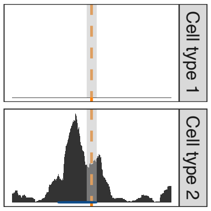	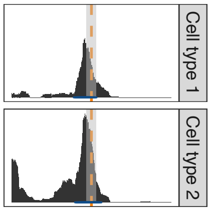
(i) Change in gene expression due to RBP knockdown: 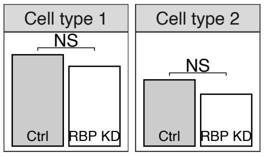	0	0	0	0
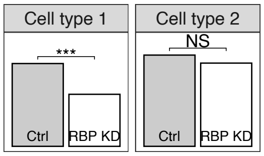	1	2	1	1
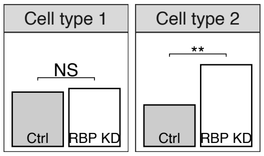	1	1	2	1
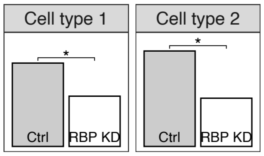	0	0	0	0

Table S2: All possible scenarios when evaluating the RBP-SNV gene impact score.

Table S3: Comparison of the sizes of the compiled epigenomic library of SF3B4 in K562 cells with the corresponding data from the ENCODE project.

File type	Output type	File size	
		ENCODE	Library
bam	alignments, replicate 1	198MB	12.4MB
bam	alignments, replicate 2	243MB	14.8MB
bed	IDR peak	229KB	
bed	peak, replicate 1	1.27MB	1.2MB
bed	peak, replicate 2	1.28MB	

to epigenomic information of variants, significantly reducing computation time for INCA, and offering a more direct view of variant-level information.

5.1 Aligned read counts from the ENCODE eCLIP-seq data collection

Table S3 showcases the reduction in file sizes when we pre-compute the epigenomic libraries. The files of aligned reads in ENCODE are typically in bam format, and as seen in Table S3, they can be quite large, with a size of approximately 200MB. Without the advantage of using this pre-computed epigenomic library, the computation time needed to aggregate aligned reads for INCA would involve two main steps: (1) loading the bam file into memory and (2) calculating normalized read counts for specific variants. To address this issue, we re-formatted the files by utilizing run-length encoding (RLE) which stores runs of genomic positions, in which the same data value occurs in many consecutive positions, as a count and single data value. For example, in the following data with the RLE format, a variant at chr1:14,700 has a normalized read count

Chr	Length	Normalized read counts
chr1	14,716	0
chr1	26	1.163137
chr1	24	0
chr1	35	1.163137

of 0 and a variant at chr1:14,767 has a normalized read count of 1.163. By adopting this approach, we gain the ability to access variant-level information in a more efficient manner. Additionally, converting the original data files into the specified format above leads to on average 16-fold reduction in required storage space (Table S3).

5.2 Peak signal value from the ENCODE eCLIP-seq data collection

ENCODE peak files are typically in bed format, comprising peaks from two replicates and IDR peaks, as seen in Table S3. INCA pre-processing excluded peaks with p-value > 0.05 and merged signal values (measurement of overall enrichment for the region) from peaks into one file as follows: A variant at chr1:16,220 inherits a signal value of 2.26 (based on a peak

Chr	Start	End	IDR	Rep1	Rep2
chr1	16,210	16,258		2.26	
chr1	16,243	16,317			2.11
chr1	16,774	16,820			4.55
chr1	17,454	17,502	4.10	4.26	

detected in Rep 1) and a variant at chr1:17,500 inherits a signal value of 4.26 (based on the composite signal value reported under the IDR column).

5.3 Differential gene expression analysis from the ENCODE shRNA RNA-seq data collection

Differential gene expression (DGE) files are directly obtained from ENCODE shRNA RNA-seq collection, consisting of 242 RBPs in HepG2 and K562 cell lines (221 in HepG2 and 201 in K562) (Luo *et al.*, 2020). These DGE files provide various fields related to testing the differences between the mean expression of the genes between the shRNA and wild type conditions, such as log2 fold change of expression, p-values from the tests. To compute the RBP-SNV gene impact score, INCA requires two specific fields: `gene` and `q-value` (or `p-value`), where the q-value represents the adjusted p-value from the false discovery rate control. In the following example, INCA identifies KLHL17 and SNORA76 as genes that exhibit significant changes in expression upon the knockdown of RBP HNRNPK in HepG2 cell line, as their q-values are less than 0.05. In contrast, AAAS and ABT1 do not show significant changes in expression.

gene name	q-value
AAAS	0.5817
ABT1	0.1810
KLHL17	0.0005
SNORA76	0.0262

Our current implementation of INCA has pre-computed eCLIP-seq library. We also developed functions and accompanying R package vignette that extend these computations for ChIP-seq data to interrogate transcription factor binding.

6 Computation time

In addition to pre-computation of epigenomic library of eCLIP-seq experiments, INCA leverages parallel computation within R and implements simultaneous execution of multiple segments within a larger computational task across multiple processing cores. On average, runtime of INCA for 15,000 variants per RBP is 10 seconds, with a maximum of 12 seconds, when executed on a computing cluster equipped with 48 CPUs and 252GB of memory. We further applied INCA to 64 sets of whole exome sequencing data (data not shown), yielding an average INCA scoring time of around 59 seconds (maximum 72 seconds) for an average of approximately 280,000 variants per RBP. Fig. S3 summarizes the computing time associated with each module across 37 RBPs.

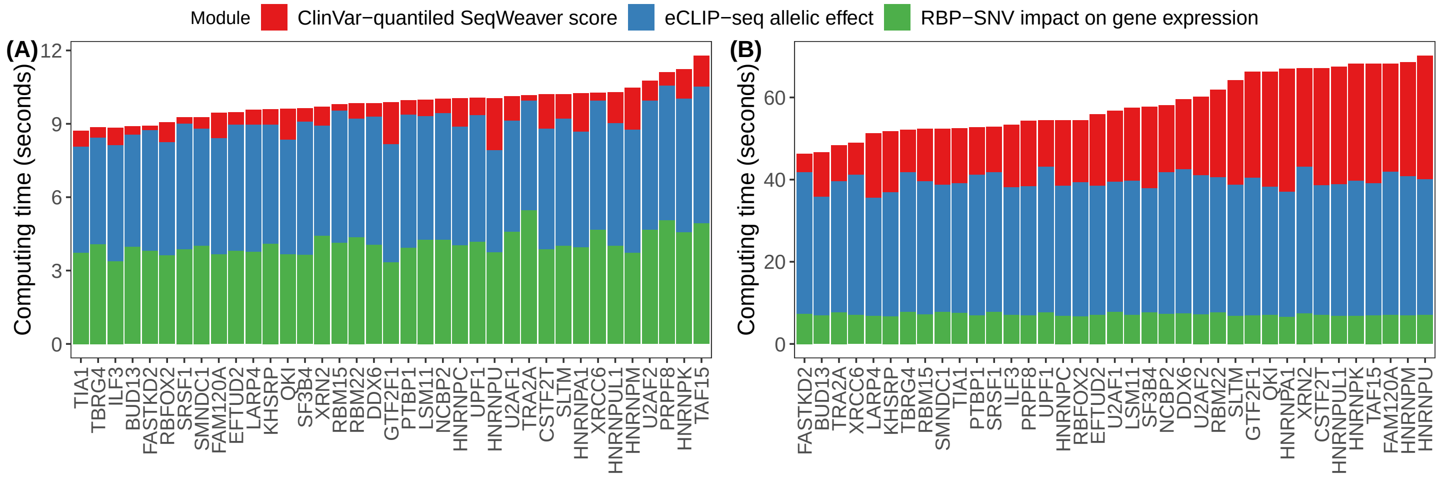

Figure S3: Module-specific computation time of INCA across 37 RBPs: (A) for ∼ 15,000 variants; (B) for ∼ 280,000 variants on a computing cluster with 48 CPUs and 252GB of memory.

7 Application with ChIP-seq data

We have demonstrated the application of INCA by leveraging a pre-computed eCLIP-seq library to assess the impact of 388 lead SNVs identified in a GWAS for TG (Graham *et al.*, 2021) on RBP activities. INCA can be extended to evaluate variants using other sequencing-based chromatin or RNA-protein interaction profiling data, such as iCLIP-seq and ChIP-seq. Here, we present another application of INCA using ChIP-seq data for the transcription factor (TF) ATF3 to evaluate the impact of these 388 SNVs on ATF3 binding. Transcription factor ATF3 is selected as an example because it regulates lipid and glucose metabolism in multiple ways, including the control of TG metabolism (Hu *et al.*, 2022).

7.1 Implementation details

The input variant file remains the same with the following fields: `Chr`, `Pos`, `Start`, `End`, `Ref`, `Alt`, and `Gene`. Similarly, INCA then derives three scores for the effect of each variant on TF activities using available cell lines (K562 and HepG2 for this example):

1. *ClinVar-quantiled DeepSEA scores:* DeepSEA (Zhou and Troyanskaya, 2015) is a deep learning-based framework trained on genome-wide chromatin profiles, including TF binding, DNase I–hypersensitive sites, and histone marks, to predict the chromatin effects of variants. Following the same computational approach for *ClinVar-quantiled SeqWeaver scores* described in Supp. Section 1, we applied DeepSEA to 125,774 pathogenic single nucleotide variants from ClinVar. INCA transforms DeepSEA scores of a variant to represent proportion of pathogenic ClinVar variants that have scores smaller than that of the given variant.

2. *Allelic effect score from ChIP-seq profiles:* We obtained IDR thresholded peak files from ENCODE (Luo *et al.*, 2020) and directly followed the setting (1) in Supp. Section 2. For each variant with an allelic difference between the cell lines (HepG2 and K562 in this example), INCA computes a score of allelic effect of TF binding. This score is set to 1 if the variant of interest resides within a binding region of the TF as defined by an IDR peak in one cell line, but not in the other cell line.

3. *TF-SNV impact on gene expression:* Currently, differential gene expression (DGE) analysis results with shRNA knockdown and wild type conditions from ENCODE (Luo *et al.*, 2020) are exclusively available for RBPs; thus, the computation of *TF-SNV gene impact score* is limited in this example. However, DGE analysis results with TF knockdown/knockout from alternative sources can be integrated into INCA as long as the data files adhere to the format specified in Supp. Section 5.3. Leveraging DGE analysis results, INCA scores the variant on whether the variant harboring gene is a direct or indirect target of the TF.

7.2 Results

Out of 388 lead SNVs from the TG GWAS (Graham *et al.*, 2021), 343 have at least one LD partner (LD R2 ≥ 0.7), resulting in a total of 14,945 SNVs of interest. INCA scores these and reveals that, on average, 3.3% of a lead SNV's LD partners receive an INCA score greater than 1 out of 2 total (Fig. S4(A)). Among the 343 lead SNVs, 48.7% have over 50% of their LD partners scoring higher than their own INCA score (Fig. S4(B)). Collectively, INCA enhanced the scoring of 3.3% of candidate variants with evidence for their impact on TF ATF3 bindings.

8 Data resources

We pre-computed an eCLIP-seq library and applied INCA to assess the impact of variants on RBP activities. INCA can be extended to evaluate variants using other sequencing-based chromatin or RNA-protein interaction profiling data, such as iCLIP-seq and ChIP-seq. In this section, we present some additional data resources that can be used within INCA.

SeqWeaver (Park *et al.*, 2021) and DeepSEA (Zhou and Troyanskaya, 2015) are pre-trained deep learning models. KnockTF (Feng *et al.*, 2023) is a gene expression profile database with knockdown/knockout of transcription (co-)factors in multiple species.

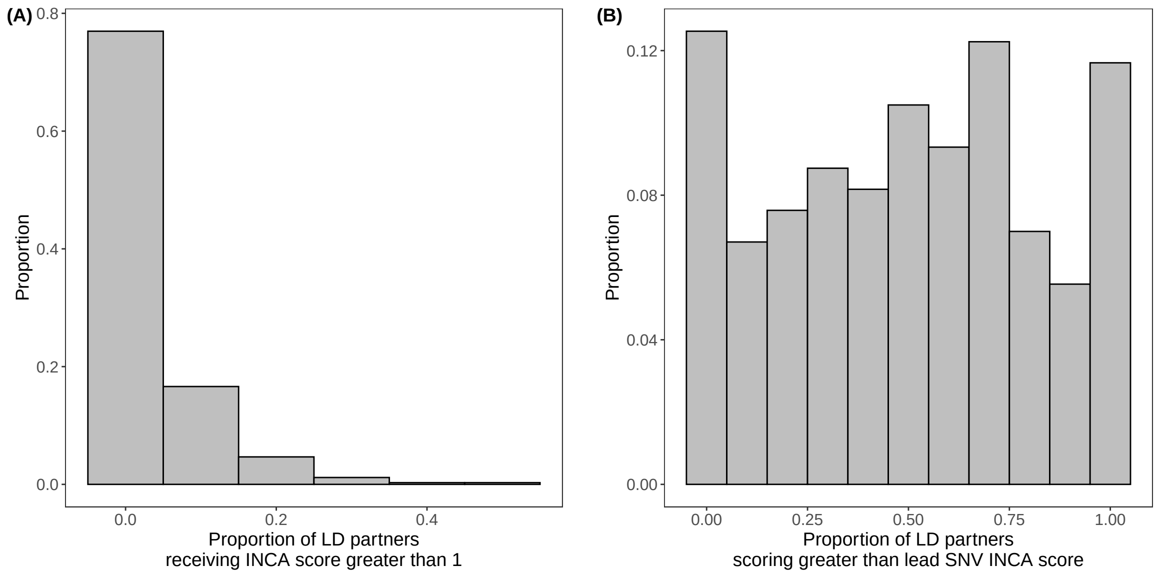

Figure S4: (A) Proportion of LD partners receiving INCA scores greater than 1 for TF ATF3. (B) Proportion of LD partners with INCA scores exceeding the score of the lead SNV for TF ATF3.

Table S4: Available data resources for INCA.

INCA score	Data resources	Summary
ClinVar-quantiled score	SeqWeaver	232 RBP models
	DeepSEA	690 TF binding profiles, 125 DHS profiles and 104 histone-mark profiles
Allelic effect score	eCLIP (ENCODE)	225 profiles, 150 different RBPs
	TF ChIP-seq (ENCODE)	2,143 profiles, 668 different TFs
	Histone ChIP-seq (ENCODE)	2,319 profiles, 33 different histone modifications
	Roadmap Epigenomics Project	3,256 profiles (including TFs and histone modifications)
Protein-SNV gene impact score	shRNA RNA-seq (ENCODE)	493 profiles
	siRNA RNA-seq (ENCODE)	24 profiles
	KnockTF	1,086 profiles (including 292 from ENCODE)

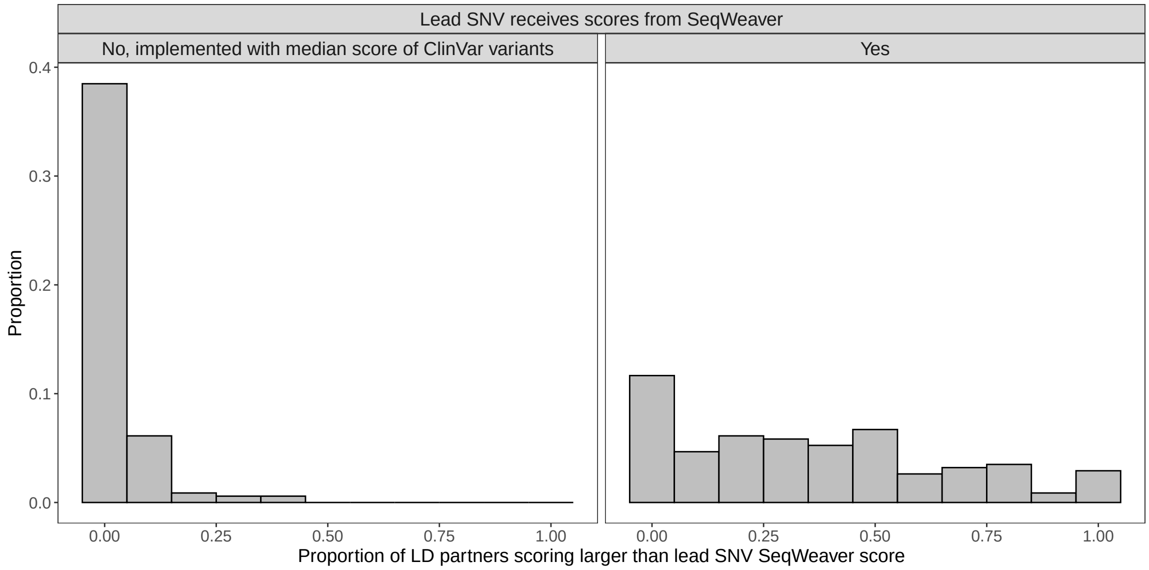

Figure S5: Proportion of LD partners with SeqWeaver scores exceeding either the score of the lead SNV or the median score of pathogenic variants in ClinVar.

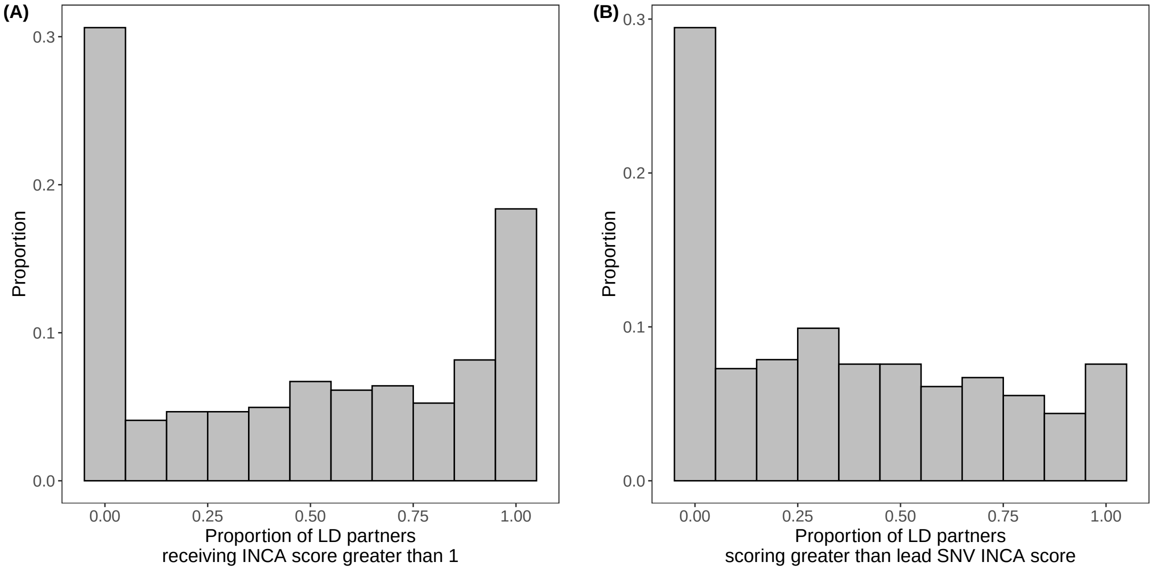

Figure S6: (A) Proportion of LD partners receiving INCA scores greater than 1. (B) Proportion of LD partners with INCA scores exceeding the score of the lead SNV.

Table S5: The variant information augmented by INCA score.

INCA score range	SeqWeaver	Allelic effect	RBP-SNV impact on gene expression: Significant change in gene expression due to RBP knockdown in cell type 1 (but not in cell type 2)	RBP peak at variant location in cell type 1 (but not in cell type 2)
0	×	×	×	×
0 - 1	✓	×	×	×
1	×	✓or	✓	×
1 - 2	✓	✓or	✓	×
2	×	✓	✓	×
2 - 3	✓	✓	✓	×
3	×	✓	✓	✓
3 - 4	✓	✓	✓	✓

References

Bailey, T. L., Johnson, J., Grant, C. E., and Noble, W. S. (2015). The MEME Suite. *Nuc. Acids Res.*, **43**(W1), W39–W49.

Cook, K. B., Kazan, H., Zuberi, K., Morris, Q., and Hughes, T. R. (2011). Rbpdb: a database of rna-binding specificities. *Nuc. Acids Res.*, **39**(suppl_1), D301–D308.

Feng, C., Song, C., Song, S., Zhang, G., Yin, M., Zhang, Y., Qian, F., Wang, Q., Guo, M., and Li, C. (2023). KnockTF 2.0: a comprehensive gene expression profile database with knockdown/knockout of transcription (co-)factors in multiple species. *Nucleic Acids Research*, **52**(D1), D183–D193.

Giudice, G., Sánchez-Cabo, F., Torroja, C., and Lara-Pezzi, E. (2016). ATtRACT-a database of RNA-binding proteins and associated motifs. *Database*, **2016**, baw035.

Graham, S. E., Clarke, S. L., Wu, K.-H. H., Kanoni, S., Zajac, G. J. M., Ramdas, S., Surakka, I., Ntalla, I., Vedantam, S., Winkler, T. W., Locke, A. E., Marouli, E., Hwang, M. Y., Han, S., Narita, A., Choudhury, A., Bentley, A. R., Ekoru, K., Verma, A., Trivedi, B., Martin, H. C., Hunt, K. A., Hui, Q., Klarin, D., VA Million Veteran Program, Global Lipids Genetics Consortium, and Willer, C. J. (2021). The power of genetic diversity in genome-wide association studies of lipids. *Nature*, **600**, 675–679.

Hitz, B. C., Lee, J.-W., Jolanki, O., Kagda, M. S., Graham, K., Sud, P., Gabdank, I., Strattan, J. S., Sloan, C. A., Dreszer, T., Rowe, L. D., Podduturi, N. R., Malladi, V. S., Chan, E. T., Davidson, J. M., Ho, M., Miyasato, S., Simison, M., Tanaka, F., Luo, Y., Whaling, I., Hong, E. L., Lee, B. T., Sandstrom, R., Rynes, E., Nelson, J., Nishida, A., Ingersoll, A., Buckley, M., Frerker, M., Kim, D. S., Boley, N., Trout, D., Dobin, A., Rahmanian, S., Wyman, D., Balderrama-Gutierrez, G., Reese, F., Durand, N. C., Dudchenko, O., Weisz, D., Rao, S. S. P., Blackburn, A., Gkountaroulis, D., Sadr, M., Olshansky, M., Eliaz, Y., Nguyen, D., Bochkov, I., Shamim, M. S., Mahajan, R., Aiden, E., Gingeras, T., Heath, S., Hirst, M., Kent, W. J., Kundaje, A., Mortazavi, A., Wold, B., and Cherry, J. M. (2023). The ENCODE Uniform Analysis Pipelines, elocation-id = 2023.04.04.535623. *bioRxiv*.

Hu, S., Zhao, X., Li, R., Hu, C., Wu, H., Li, J., Zhang, Y., and Xu, Y. (2022). Activating transcription factor 3, glucolipid metabolism, and metabolic diseases. *Journal of Molecular Cell Biology*, **14**(10), mjac067.

Luo, Y., Hitz, B. C., Gabdank, I., Hilton, J. A., Kagda, M. S., Lam, B., Myers, Z., Sud, P., Jou, J., Lin, K., Baymuradov, U. K., Graham, K., Litton, C., Miyasato, S. R., Strattan, J. S., Jolanki, O., Lee, J.-W., Tanaka, F. Y., Adenekan, P., O'Neill, E., and Cherry, J. M. (2020). New developments on the encyclopedia of dna elements (encode) data portal. *Nuc. Acids Res.*, **48**(D1), D882–D889.

Park, C. Y., Zhou, J., Wong, A. K., Chen, K. M., Theesfeld, C. L., Darnell, R. B., and Troyanskaya, O. G. (2021). Genome-wide landscape of rna-binding protein target site dysregulation reveals a major impact on psychiatric disorder risk. *Nat Genet*, **53**(2), 166–173.

Paz, I., Kosti, I., Ares, Jr, M., Cline, M., and Mandel-Gutfreund, Y. (2014). RBPmap: a web server for mapping binding sites of RNA-binding proteins. *Nuc. Acids Res.*, **42**(Web Server issue), W361–W367.

Ray, D., Kazan, H., Cook, K. B., Weirauch, M. T., Najafabadi, H. S., Li, X., Gueroussov, S., Albu, M., Zheng, H., Yang, A., Na, H., Irimia, M., Matzat, L. H., Dale, R. K., Smith, S. A., Yarosh, C. A., Kelly, S. M., Nabet, B., Mecenas, D., Li, W., Laishram, R. S., Qiao, M., Lipshitz, H. D., Piano, F., and Hughes, T. R. (2013). A compendium of RNA-binding motifs for decoding gene regulation. *Nature*, **499**, 172–177.

Van Nostrand, E. L., Pratt, G. A., Yee, B. A., Wheeler, E. C., Blue, S. M., Mueller, J., Park, S. S., Garcia, K. E., Gelboin-Burkhart, C., Nguyen, T. B., Rabano, I., Stanton, R., Sundararaman, B., Wang, R., Fu, X.-D., Graveley, B. R., and Yeo, G. W. (2020). Principles of RNA processing from analysis of enhanced CLIP maps for 150 RNA binding proteins. *Genome Biology*, **21**.

Zhou, J. and Troyanskaya, O. G. (2015). Predicting effects of noncoding variants with deep learning–based sequence model. *Nature methods*, **12**(10), 931–934.

Zuo, C., Shin, S., and Keleş, S. (2015). atSNP: transcription factor binding affinity testing for regulatory SNP detection. *Bioinformatics*, **31**(20), 3353–3355.
